# Moxibustion for Primary Dysmenorrhea: An Adjuvant Therapy for Pain Relief

**DOI:** 10.1155/2022/6864195

**Published:** 2022-01-27

**Authors:** Sian Pan, Shaohua Wang, Juan Li, Hanyu Yuan, Xiao Xue, Yu Liu, Zenghui Yue

**Affiliations:** College of Acupuncture, Massage and Rehabilitation, Hunan University of Chinese Medicine, Changsha 410208, China

## Abstract

The latest spectrum of moxibustion disease shows that primary dysmenorrhea is a high-frequency symptom of moxibustion and that it is the dominant clinical disease. In the specific treatment methods, all types of moxibustion methods have been widely used, such as thermal, thunder fire, partitioned, and spreading moxibustion. Moxibustion plays a therapeutic role through its four mechanisms of action: heat, light, moxa smoke, and drug effects. The mechanism of moxibustion treatment for primary dysmenorrhea focuses on adjusting endocrine hormones, regulating immune function and neuro-related factors, and improving uterine microcirculation. In this study, based on the clinical evidence of different moxibustion methods for treating primary dysmenorrhea, the design model, intervention characteristics, and clinical outcomes were analyzed. Meanwhile, the brain effect mechanisms of different imaging methods were summarized from the perspective of neuroimaging. It was pointed out that the left anterior cingulate gyrus, left inferior parietal angular gyrus, and left superior gyrus may be the analgesic brain regions that regulate sensory, emotional, and cognitive aspects. Moreover, the neural circuits involved can be inferred: the frontal cortex-basal ganglia (the pea nucleus)-cerebral cortex, which mediates motivation and emotional drive, and the parietal lobe-basal ganglia-limbic lobe-frontal lobe, which is involved in neurotransmitter transport and emotional regulation and behavioral expression. There are still problems and deficiencies in studies on the mechanism of moxibustion treatment for primary dysmenorrhea. Studies should be strengthened on how moxibustion produces an effect. Attention should be paid to exploring how the spectrum range and peak in the light effect of moxibustion treat primary dysmenorrhea. Studies assessing the mechanisms of moxibustion treatment for primary dysmenorrhea should be conducted to provide an experimental basis and evidence-based medical evidence for clinical treatment.

## 1. Introduction

Primary dysmenorrhea (PD) is a functional disease characterized by intermittent lower abdominal pain before and after menstruation, without apparent pelvic organic lesions. At the onset of the disease, a patient experiences intermittent pain in the lower abdomen, which can radiate to the lumbosacral portion. Patients with severe PD may experience nausea, vomiting, cold hands and feet, sweating, and even syncope, seriously affecting their quality of life [1]. Reports of the epidemiological prevalence of PD vary widely across studies, ranging from 17% to as high as 90%, with more than half of women describing their pain as moderate to severe. Long-term PD can lead to repression, depression, agony, and other destructive emotions. However, the pathogenesis of PD is complex, and its pathological mechanism remains unclear. Moreover, the current treatment for PD can only alleviate the symptoms in some patients [2]. Currently, nonsteroidal anti-inflammatory drugs (NSAIDs) seem to be effective in treating PD, but their adverse reactions also pose some health risks [3, 4]. Thus, determining a safe and effective therapeutic approach for patients with PD is imperative. Xu et al. [5] performed a meta-analysis involving 20 randomized controlled trials (RCTs) with a sample size of 2134 cases. A comparison of the therapeutic effects of moxibustion, acupuncture, floating eye of the needle, and point application with other treatment methods in the treatment of PD proved that moxibustion combined with point therapy has an overall curative effect on PD. The results of the meta analysis showed that: Firstly, the total efficacy for the 2 studied interventions was better, with a statistically significant difference from that of the control methods: degrees of freedom (d*f*) = 14, relative risk (RR) = 1.19, 95% confidence interval (95% CI) = (1.14–1.24), *P* < 0.000 for the UTG, and d*f* = 4, RR = 1.15,95% CI (1.02–1.29), *P*=0.03 for the CDSTG; secondly, the studied interventions were better than the control methods, with statistically significant differences, in relieving the severity of symptoms of PD: d*f* = 3, mean difference (MD) = 3.20, 95% CI (2.36–4.04), *P* < 0.000 for the UTG and d*f* = 1, MD = 2.09, 95% CI (0.16–4.02), *P*=0.03 for the CDSTG; third, no statistical difference existed between the intervention and control methods groups in the reduction of the level of peripheral blood PGF2*α*: d*f* = 2, standardized mean difference (SMD) = 0.13, 95% CI (−0.13–0.39), *P*=0.32. Gou et al. [6] extracted data for studies searched from 10 electronic databases, evaluated the methodological quality of the included studies, and discussed three outcomes: effective rate, pain remission, and prostaglandin F2*α* (PGF2*α*) level in serum. Current clinical studies have shown that, compared with nonmoxibustion treatments for PD, moxibustion leads to a higher effective rate and lower level of PGF2*α* in serum (MD = −4.65,95% CI (−8.42, −0.88), *P*=0.02). Moreover, research on the disease spectrum and indications of moxibustion therapy based on bibliometric analysis shows that PD is the dominant disease of moxibustion [7, 8].

Moxibustion has a history of thousands of years in China. It originated after humans mastered fire, and countless clinical practices have confirmed its therapeutic effect. Moxibustion treats diseases by burning moxa. The thermal product of burning is an essential factor for producing a therapeutic effect [9]. Additionally, modern medicine believes that hyperthermia has several clinical effects. First, hyperpyrexia can heat local tissues; strengthen metabolism and enzyme reactions; dilate microvessels, thereby strengthening automatic congestion and enhancing phagocytosis; treat local subacute and chronic inflammation. Second, it can reduce the excitability of nerve endings; relieve neuralgia, myalgia, and arthralgia and have antispasticity effects.

Certain bacteria that are not resistant to heat can be reduced, thus achieving the purpose of sterilization. Finally, it can strengthen perspiration, promote metabolism, improve nutrition, and stimulate cell growth and regeneration. More importantly, modern studies on moxibustion have shown that these effects are within the scope of warm moxibustion efficacy. In summary, the thermogenetic effect of moxibustion can not only affect the dynamic distribution of temperature field in biological tissue under moxibustion and the macroscopic energy (thermal energy) migration and change process caused by moxibustion heat but also stimulate the spontaneous infrared spectrum change of thermally sensitive acupoints to produce “resonance.” Simultaneously, the combustion products of active ingredients in Folium *Artemisia argyi* can also play an auxiliary therapeutic role. Generally, the organic combination of meridians and acupoints with the physical and chemical effects of moxibustion produces the “comprehensive effect” of moxibustion.

Moxibustion treatment for PD is closely related to central analgesia. A large amount of objective visual evidence provided by neuroimaging indicates that moxibustion treatment of PD is a comprehensive process of treating diseases by stimulating acupoints to regulate the brain network effect [10, 11]. A previous study assessed the central analgesic mechanism of moxibustion in the treatment of PD before and after moxibustion by observing resting-state functional magnetic resonance imaging (rs-fMRI) of the brain [12]. Therefore, this paper will review the mechanism of moxibustion in the treatment of PD and the research progress of moxibustion and neuroimaging for reference.

## 2. Pathophysiological Studies

**Figure 1:**
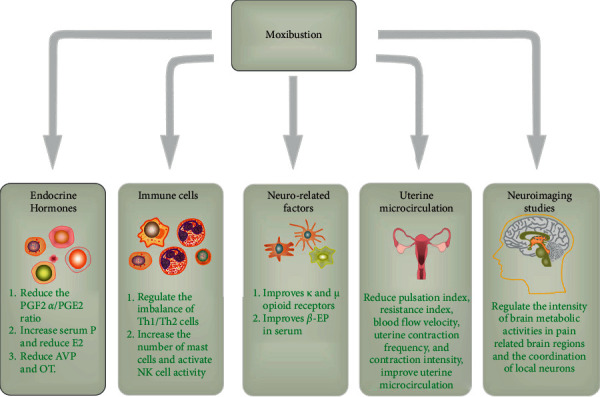
Pharmacological mechanism of moxibustion in the treatment of primary dysmenorrhea. PGE2, prostaglandin E2; PGF2*α*, prostaglandin F2*α*; P, progesterone; E2, estradiol; AVP, vasopressin; OT, oxytocin; *β*-EP, *β*-endorphin.

The mechanism of moxibustion in the treatment of PD is mainly focused on regulating endocrine hormones, immune function, and nerve factors and improving uterine microcirculation (Figure 1).

### 2.1. Endocrine Hormones

#### 2.1.1. Prostaglandin E2 and Prostaglandin F2*α*

Abnormal uterine contractions in patients with PD and nausea, vomiting, and diarrhea in over 60% of patients with PD are associated with prostaglandin (PG). PGE2 and PGF2*α* are closely related to PD [13]. PGF2*α* can stimulate the contraction of arcuate vessels and lead to local hypoxia in the endometrial tissue. Endometrial PGF2*α* can increase the contraction and tension of the uterine smooth muscle. A high concentration of PGF2*α* acts on the PGF2*α* receptor on the wall of the spiral arterioles, causing spasmodic contraction of the uterine smooth muscle.

In comparison, a low concentration of PGE2 can inhibit the spontaneous activity of the uterine smooth muscle. The gradual increase in the PGF2*α*/PGE2 ratio results in excessive contraction of uterine smooth muscle, ischemia, and hypoxia, increasing the sensitivity of peripheral nerves to pain and causing dysmenorrhea [14]. The imbalance of PGE2 and PGF2*α* secretion is considered the leading cause of PD. Moreover, this process is accompanied by the accumulation of acidic metabolites and a decrease in the pain threshold [15].

Various moxibustion methods have confirmed that the mechanism of moxibustion in the treatment of PD is closely related to the regulation of PGE2 and PGF2*α* levels [16]. Panbi et al. [17] treated 40 Wistar dysmenorrhea model rats with moxa stick moxibustion, which confirmed that moxa moxibustion could alleviate dysmenorrhea symptoms and downregulate PGF2*α* content in uterine tissue and improve hemorheology (whole blood viscosity, plasma-specific viscosity, erythrocyte electrophoresis time). Huijuan and Mingxin [18] found in the comparative clinical observation of thunder-fire moxibustion combined with ear points and NSAIDs in 76 patients with PD that the treatment group had significantly reduced PGF2*α*/PGE2 ratio and that the treatment relieved the clinical symptoms of patients with PD. Sen and Dongfu [19] used moxa stick mild moxibustion at Guanyuan (GV4) point to treat 60 patients with PD and confirmed that moxibustion could reduce the serum PGF2*α*/PGE2 ratio. Limei et al. [20], Xin et al. [21], and Wu [22] treated patients with PD with ginger-partitioned moxibustion and confirmed that it could reduce the serum PGF2*α* level in patients with PD and relieve clinical symptoms. Jia and Li [23] treated 80 patients with PD with cold coagulation and blood stasis syndrome with thunder-fire moxibustion at the Shenque (CV8) and Guanyuan (GV4) points. It was proven that thunder-fire moxibustion could reduce serum PGF2*α* level and relieve dysmenorrhea symptoms.

#### 2.1.2. Estradiol and Progesterone

Estradiol (E2) is an estrogen; there are two different peaks of estrogen before and after ovulation, which can increase the PG level in the endometrium before menstruation. In contrast, progesterone (P) can antagonize this effect, reduce the production of PG, and decrease the contraction activity of the uterine smooth muscle to alleviate dysmenorrhea. E2 and P act antagonistically in the uterus. When P level increases, it inhibits the synthesis of PGF2*α*, promotes blood circulation, stimulates the production and release of analgesic substances (such as *β*-endorphin [*β*-EP]), and reduces uterine smooth muscle spasms, thereby relieving dysmenorrhea symptoms. However, when the E2 level increases, it promotes the secretion of PGF2*α*, aggravates uterine contraction, and causes dysmenorrhea [24].

Moxibustion can effectively treat PD by increasing serum P and decreasing E2 levels. Fan [25] found that partitioned moxibustion increased the expression of P and reduced the expression of E2 in dysmenorrhea rats. Partitioned moxibustion on the umbilicus [26, 27] and electronic moxibustion [28] were selected to treat patients with PD, and the same conclusion was obtained. It was confirmed that partitioned moxibustion on the umbilicus and electronic moxibustion could reduce serum E2 level, increase P level, and relieve dysmenorrhea symptoms. At the same time, it was found that the long-term efficacy of the electronic moxibustion group in the treatment of PD was better than that of the NSAIDs group. A previous study [29] showed that drug-paste-separated moxibustion of Guanyuan (GV4) effectively relieves pain in PD rats, which is probably associated with its effects in downregulating serum E2 level and endometrial ER mRNA expression and upregulating serum P and endometrial PR mRNA expression levels.

#### 2.1.3. Vasopressin and Oxytocin

Vasopressin (AVP) is a hormone secreted by the pituitary gland. Its secretion changes with the concentration of estradiol during the menstrual cycle. The concentration of AVP is lower in the follicular phase and increases during the ovulation phase. AVP acts as a catalyst for PG synthesis, promoting PG synthesis and reducing uterine blood flow caused by pain. AVP may increase uterine contractions and reduce blood flow through the uterus, leading to ischemia and dysmenorrhea [30, 31]. Several researchers have demonstrated the role of AVP in the pathological mechanism of dysmenorrhea [32]. Studies have shown that AVP is a more potent uterine contraction agent than oxytocin (OT) and strongly stimulates the nonpregnant uterus. OT mainly excites the smooth muscle and uterine artery of the uterus, resulting in the acceleration of uterine contraction; simultaneously, phosphate is activated to stimulate intimal cells to release PGs, which aggravates dysmenorrhea. AVP and OT can contract uterine blood vessels, resulting in temporary uterine ischemia and dysmenorrhea [33].

Moxibustion can alleviate dysmenorrhea symptoms by reducing AVP and OT levels. Ye et al. [34] confirmed that herb-partitioned moxibustion at the Mingmen (GV4) acupoint in treating dysmenorrhea model rats could reduce the expression of OT and AVP (*P* < 0.05) and simultaneously regulate the expressions of PGF2*α*, PGF2*α*-R, PGE2, and PGE2-R (*P* < 0.05). Zhenzhen et al. [35] treated dysmenorrhea model rats with moxibustion at Sanyinjiao (SP6). The results showed that moxibustion significantly reduced the peak, mean, and lane values of uterine OT (*P* < 0.01). Wei and Hu [36] used thermal moxibustion at the Guanyuan (CV4) point to treat 117 patients with PD with cold coagulation and dampness stagnation and confirmed that thermal moxibustion could reduce AVP and PGF2*α* levels in menstrual blood and increase the range of PGE2.

### 2.2. Immune Function

#### 2.2.1. Th1/Th2 Cell Balance

Th1 and Th2 cells play different roles in immunity and disease. Th1 cells can effectively stimulate and mediate cellular immunity and cytotoxic T cells, activate macrophages, result in delayed hypersensitivity, and eliminate infection caused by intracellular parasitic bacteria. Nevertheless, Th2 cells stimulate humoral immunity. Interleukin-1 (IL-4) secreted by Th2 cells can promote the proliferation of B cells and induce the production of antibodies, especially immunoglobulin E (IgE) [37]. Acupuncture can also regulate the differentiation of different subsets of T cells; that is, it maintains the balance of T cells (Th1/Th2, Th17/Treg) and regulates Th1/Th2 and Th17/Treg to drift to Th2 and Treg, respectively. Thus, this inhibits the production of inflammatory factors (IL-2, IL-12, and interferon [IFN]-*γ*), increases the expression of anti-inflammatory factors (IL-10), and reduces substances, such as antibody IgE, leukotriene B4, and nitric oxide in serum [38, 39], affecting inflammatory signaling molecules (extracellular signal-related kinase, nuclear factor kappa B, activator protein 1, p38).

Moxibustion can regulate the immune function of patients with PD, reduce the level of Th1 cytokine IFN-*γ*, promote the expression of serum Th2 cytokines IL-4 and IL-10, regulate the imbalance of Th1/Th2 cells, promote the recovery of immune function, inhibit the production of inflammatory cytokines, continue to play an analgesic role, and relieve the symptoms of dysmenorrhea [40]. Yanna [41] used ginger-partitioned moxibustion to treat 92 patients with PD and found that moxibustion at Sanyinjiao (SP6) could increase the expression of sera IL-4 and IL-10, continuously play an analgesic role, and improve the therapeutic effect. Chen et al. [42] and Le et al. [43] confirmed that medicine-partitioned moxibustion at the Shenque (CV8) point could reduce plasma IFN-*γ* and increase plasma IL-4 and IL-10 levels to restore the balance of immune function in patients with PD.

#### 2.2.2. Mast Cell and Natural Killer Cell

Early studies have shown mast cell aggregation in small blood vessels, tiny nerves, and nerve endings along the meridian in humans and rats. Acupuncture stimulation can significantly increase the aggregation and degranulation of mast cells along the meridian. Acupuncture and moxibustion can bidirectionally regulate the function of mast cells; that is, it can alleviate the abnormal degranulation of mast cells in the pathological state [44]. Recent studies have shown that substances released by mast cells (adenosine triphosphate, exosomes) can act on local nerve endings and form “synaptic-like” connections with neurons [45, 46]. Small molecule signaling substances (microRNA-155) and cytokines (IL-33) derived from mast cells produce “neuromodulator” effects and regulate “synaptic” functions. Acupuncture and moxibustion alleviate mast cell degranulation and promote “synaptic like” function, which is an essential immune mechanism for the treatment of allergic diseases (asthma) and inflammatory diseases (inflammatory pain) [47, 48]. Natural killer (NK) cells are a class of lymphocytes that lack antigen-specific cell-surface receptors. Acupuncture and moxibustion have bidirectional regulatory effects on NK cells. Acupuncture and moxibustion can increase the number of NK cells, enhance NK cell activity in low immunity (such as chronic stress and fatigue syndrome), and promote the secretion of immune factors by NK cells (such as IFN-*γ*, IL-10, granulocyte-macrophage stimulating factor) [49, 50]. NK cells are critical immunomodulatory cells in the body and prominent lymphocytes in the endometrial tissue. Studies have shown that activating the activity of NK cells can enhance the pain threshold [51].

Moxibustion can relieve dysmenorrhea symptoms by increasing the number of mast cells and activating NK cell activity. Zhongyin et al. [52] observed the effect of herb-partitioned moxibustion at Guanyuan (CV4) on NK cell activity in rats with PD and confirmed that herb-partitioned moxibustion at Guanyuan (CV4) could increase the activity of uterine NK cells and reduce uterine PGF2*α* level, and the effect of herb-partitioned moxibustion on NK cell activity in PD rats was better than that of direct moxibustion. Li et al. [53] treated 50 female Wistar dysmenorrhea model rats with cold coagulation and blood stasis by medicine-partitioned moxibustion at the Shenque (CV8) point. The results showed that it could increase the number of mast cells in the Shenque (CV8) point area of dysmenorrhea model rats and promote their degranulation to alleviate the symptoms of dysmenorrhea. Qi et al. [54] found that moxibustion at the Guanyuan (CV4) and Shenque (CV8) points could exert analgesic effects by increasing the activity of NK cells in PD rats.

### 2.3. Neuro-Related Factors

The effect of moxibustion on PD is closely related to its central analgesic mechanism. Opioid receptors are widely distributed in the central nervous system and play an essential role in the analgesic effect. Opioid receptors are coupled with G protein, inhibit the activity of adenylate cyclase and calcium channel, activate potassium channel, and prevent the transmission of pain impulse, resulting in analgesic effect. The *κ* and *µ* receptors play an important role in central analgesia [33].

Studies have confirmed that the central analgesic mechanism of moxibustion in the treatment of PD may be related to the levels of *κ* and *µ* opioid receptors. Zhenzhen et al. [55] found that moxibustion at the Sanyinjiao and Guanyuan points in dysmenorrhea rats with cold coagulation syndrome could increase the expression level of brain *κ* receptor mRNA, significantly reducing the writhing reaction of dysmenorrhea rats with cold coagulation syndrome, and relieve uterine smooth muscle spasm. Jin et al. [56] confirmed that moxibustion at the Guanyuan point could improve the expression of cold coagulation dysmenorrhea rat spinal cord *µ* receptors, resulting in an analgesic effect.

Meanwhile, moxibustion can also exert its endogenous analgesic effect by regulating the level of *β*-EP in serum. Researchers [17, 57] found that herb-partitioned moxibustion on the navel used to treat dysmenorrhea model rats could increase the scope of plasma *β*-EP and regulate PGF2*α* and PGE2 levels in uterine tissue, improving the activity of NK cells. In treating patients with PD with moxibustion, prior studies [58, 59] reported that it could increase *β*-EP levels and regulate serum PGF2*α* and PGE2 levels, subsequently treating PD.

### 2.4. Uterine Microcirculation

Improving uterine microcirculation by moxibustion may be one of the mechanisms for relieving PD symptoms. Various moxibustion methods have been confirmed to reduce the pulsation index, resistance index (RI), blood flow velocity, uterine contraction frequency, and contraction intensity; improve uterine microcirculation; relieve dysmenorrhea symptoms [60]. Qin et al. [61] and Liu et al. [62] confirmed that warm acupuncture could significantly reduce the blood flow velocity of uterine arteries at all levels in patients with PD and improve uterine blood flow.

An RCT by Chunxin et al. [63], with a sample size of 101 cases, observed changes in the RI and pulsatility index of the uterine and arcuate arteries before and after treatment, which was detected by color Doppler ultrasound. It is concluded that moxibustion can improve the clinical symptoms of patients with PD with cold dampness stagnation and that the curative effect is better than that of meloxicam tablets. Its product is related to the improvement of the blood supply to the uterus.

Mild moxibustion [64] at the Sanyinjiao (SP6) and Guanyuan(CV4) acupoints may relax uterine microvascular obstacles by reducing PGF2*α* level in the uterine tissue, improve microcirculation disorder, and alleviate uterine swelling in PD rats. Mild moxibustion can enlarge the microvessels, improve microcirculation disturbance, and relieve uterine swelling in PD rats. During the gentle moxibustion intervention, PGF2*α* and PGE2 levels in the uterus synchronously increased or decreased, and the changes in PGE2 were noticeable, but the changes in uterine microvasculature and morphology caused by the reduction of PGF2*α* were more significant than those of PGE2.

In addition, few studies have confirmed that moxibustion can affect the expression of transient receptor potential vanilloid type channel (TRPV) receptors in the uterus, thereby affecting Ca^2+^ channels and alleviating dysmenorrhea symptoms. Chunjing and Ma [65] used umbilical therapy to treat dysmenorrhea model rats with cold coagulation and blood stasis and confirmed that umbilical treatment could reduce the expression of TRPV receptor in the uterus of dysmenorrhea model rats with cold coagulation and blood stasis, thereby inhibiting Ca^2+^ influx caused by mast cell activation, reducing the contraction of uterine smooth muscle, and achieving the therapeutic effect.

## 3. Neuroimaging Studies

In addition, some studies using rs-fMRI found that moxibustion played a central analgesic mechanism by regulating the intensity of brain metabolic activities in pain-related brain regions and the coordination of local neurons in brain regions.

### 3.1. Functional Resonance Magnetic Imaging

An RCT [66] with a sample size of 19 cases combined arterial spin labeling and fMRI with clinical efficacy evaluation (visual analog scale [VAS] score). On the first to the third day of the menstrual period, VAS score >4 points, that is, pain scale >40 mm, an immediate (30 min) moxibustion Guanyuan (CV4) or placebo moxibustion Guanyuan intervention was performed. fMRI scanning was performed before and after the intervention, and the VAS scores were recorded before and after treatment. In conclusion, first, moxibustion has a short-term analgesic effect on PD. Second, among the eight brain regions with significantly increased regional cerebral blood flow (CBF) in this study, excluding the activation of the lenticular nucleus, which had no effect on the efficacy of moxibustion in the treatment of PD, the seven brain regions (left anterior cingulate gyrus and collateral cingulate gyrus, left posterior cingulate gyrus, angular gyrus, left inferior parietal angular gyrus, superior marginal gyrus, left medial frontal gyrus, and left middle frontal gyrus) may regulate pain from three different aspects of sensation, emotion, and cognition, which is the possible mechanism of moxibustion for short-term analgesia in the treatment of PD. However, the interaction between activated brain regions requires further study. Third, the brain regions involved in analgesia are the left anterior and paracingulate gyrus, left inferior parietal angular gyrus, and left superior marginal gyrus. Fourth, there are two circuits involved in moxibustion for PD pain relief. The first is the frontal cortex of the basal ganglia cerebral cortex. Among these, the lenticular nucleus is the most critical component. This circuit mediates motivation and emotional drive to promote the expression of goal-directed behavior. The second is the parietal basal ganglia marginal frontal lobe, which is involved in neurotransmitter transport, emotion regulation, and behavior expression.

An RCT by Zhang [67], with a sample size of 24 cases, performed moxibustion at the Guanyuan (CV4) point as the research object. Seven days before menstruation was selected as the intervention time for moxibustion, and three menstrual cycles were considered a complete course of treatment. The VAS scores of subjects before and after treatment were recorded, and fMRI scans were performed before and after the intervention. First, the results showed that moxibustion had a long-term analgesic effect on PD. Second, moxibustion can alleviate anxiety caused by PD to a certain extent. Third, the brain regions with increased differences in the regional homogeneity (ReHo) values before and after treatment were the right fusiform gyrus, left angular gyrus, and left dorsolateral superior frontal gyrus. The decreased regions were the right paracentral lobule, left superior orbital frontal gyrus, left inferior occipital gyrus, left middle temporal gyrus, and right middle occipital gyrus. Fourth, the brain regions with increased CBF differences before and after treatment were the right middle frontal gyrus, left middle occipital gyrus, anterior cingulate, left paracingulate gyrus, and right precentral gyrus. In contrast, the decreased regions were the right inferior temporal gyrus, right precuneus, right caudate nucleus, left supramarginal gyrus, right superior temporal gyrus, and right superior occipital gyrus. The analysis of ReHo and CBF in the abovementioned pain-related brain areas suggests that this may be the main target of moxibustion in PD treatment.

Dingyi et al. [68] conducted a mechanism study with a sample size of 60 cases. This study performed the moxibustion method using heat-sensitive acupoints, grouped according to whether the heat-sensitive phenomenon occurred at the Guanyuan (CV4) point during moxibustion, and rs-fMRI data were collected before and after moxibustion. DPARSF software and brain functional connectivity analysis with the prefrontal lobe cortex as the seed point were used for postprocessing. Moxibustion at the thermal-sensitive Guanyuan (CV4) point can change the brain operational connection network of patients with PD. The emergence of thermal moxibustion may enhance the connection between the left brainstem and left cerebellum, inhibiting the functional relationship between the left brain white matter area insular lobe and frontal lobe and indirectly affecting the function of other brain areas related to the limbic system.

In conclusion, the application of rs-fMRI technology to study the corresponding mechanism of the brain provides an objective visual basis for clinical application and promotion. From the above, clearly, moxibustion has an analgesic effect in the short term and has an excellent long-term analgesic effect. Moreover, the left anterior and paracingulate gyrus, left inferior parietal marginal angular gyrus, and left superior marginal gyrus were analgesic brain areas and regulated pain from three different aspects: sensation, emotion, and cognition. It provides an objective reference for studying the central analgesic mechanism of moxibustion in the treatment of PD.

### 3.2. Low-Cost Temperature Transition Mixtures

Yingjie and Ye [69] conducted a mechanism study with a sample size of 60 cases. With the help of TTM technology, the patients were scanned before and after Guanyuan (CV4) point moxibustion. By observing the changes in metabolic heat form and heat radiation value in the local area of the patients, the patients were compared before and after themselves. The results showed an evident decrease in cell metabolic heat in the uterine size of patients with cold cell palace deficiency, and there was no abnormal heat source in the lumbosacral and Shenshu (BL23) points. The results objectively analyzed and explained that moxibustion at the Guanyuan (CV4) point could effectively improve the symptoms of patients with PD with cell palace deficiency and cold syndrome. This provides strong evidence for the application of TTM technology in the evaluation of PD efficacy.

## 4. Clinical Research

Our previous study showed that moxibustion could adjust the function of the viscera, promote metabolism, and enhance immune function, especially in the treatment of chronic and complex diseases and preventive healthcare. The warm effect generated by moxibustion in treating conditions is the key to achieving a curative effect [70, 71]. The mechanism of moxibustion treatment for PD focuses on adjusting endocrine hormones, regulating immune function and neuro-related factors, and improving uterine microcirculation. In the specific treatment methods, all types of moxibustion methods have been widely used, such as thermal, thunder-fire, partitioned, and spreading moxibustion. Fifty-six RCTs with 5550 patients were included in a systematic review and network meta-analysis, comparing six object-separated moxibustion therapies with acupuncture or oral medicine. Mild moxibustion can not only effectively treat PD but also relieve pain in comparison with ibuprofen, which seems to be an advisable option for PD treatment to relieve symptoms [72].

Moxibustion therapy can reduce the dose of Western medicine and any related adverse reactions. As an adjuvant therapy, moxibustion has a long-term effect on the treatment of PD. The representative clinical studies are presented in Table 1.

## 5. The Mechanism of Moxibustion

Studies have found that the mechanism of moxibustion is mainly reflected in thermal effect, light effect, moxa smoke, and drug effect. The thermal product is the primary way for moxibustion to play a therapeutic role, and moxa smoke and partition moxibustion also play an essential role in the treatment. However, there is no conclusion on the infrared radiation spectrum range and wave peak in the light effect of moxibustion [83].

### 5.1. Thermal Effect

The thermal effect is one of the most critical effects of moxibustion [84, 85]. The heat of moxibustion increases the skin's surface temperature at the moxibustion site and simultaneously reaches the muscular layer under the skin. The local temperature of the moxibustion acupoint is increased, and the specific receptors, heat-sensitive immune cells, and heat shock proteins (HSPs) in the acupoint are activated to initiate the warming effect of moxibustion and induce a variety of local effects. Through the nerve and body fluid pathways, the warm and heat stimulation signals and subsequent consequences of moxibustion are transmitted to remote organs and the whole body, causing the following effects of distant specific target organs [86]. TRPV mediates thermal pain, in which TRPV1 can be activated by moxibustion temperature above 43°C, which exists in primary sensory afferent nerve endings, endothelial cells, mast cells, and skin keratinocytes and produces a cascade reaction of biological effects through the nerve fiber pathway [87, 88]. TRPV2 can be activated by moxibustion temperature above 52°C to promote the production of active substances, such as growth factors and inflammatory regulatory factors, to produce subsequent effects [89]. When the moxibustion temperature exceeds 45°C, noxious stimulation will occur, and the harmful sensor of C fiber is activated, resulting in local thermal pain, burning pain, and other sensations and inducing a local burning reaction [90, 91]. At this time, the activation of dorsal reticular nucleus neurons in the medulla oblongata related to analgesia was suddenly strengthened, and the analgesic effect of thermal moxibustion was exerted [87]. Heat stimulation of local moxibustion can change the morphology and quantity of heat-sensitive immune cells and produce an immune response. In addition, studies have confirmed that heat stress can activate HSP and increase HSP expression. HSP is believed to be a local initiation mechanism involved in the warming effect of moxibustion, and it may be an essential substance linking the local and overall effects of moxibustion [90]. The activation of HSP70 expression may be one of the reasons for its analgesic effect [92]. More importantly, the analgesic effect caused by the activation of temperature receptors and nociceptors and the increase in HSP expression may be one of the reasons why moxibustion plays a role in the treatment of PD.

### 5.2. Light Effect

In addition to the thermal effect, moxibustion also plays a role through the light effect. Although the spectral range and wave peak produced by moxibustion combustion have not been determined, they are considered to be mainly infrared radiation [93, 94]. Far-infrared radiation provides a nondrug alternative therapy to reduce inflammation with PG and cyclooxygenase as targets, especially in the 4–14 m bands [95]. Infrared radiation is an invisible electromagnetic wave that is adjacent to the visible light region. Infrared radiation is divided into near-infrared radiation (0.8∼1.5 m), medium infrared radiation (1.5∼5.6 m), and far-infrared radiation (5.6∼1000 m) [96]. Some researchers [97] believe that the spectrum of moxibustion is mainly the far-infrared spectrum close to the near-infrared region, but others [98] believe that it belongs to the near-infrared radiation with strong penetration and can penetrate deep tissue below the *epidermis*. In addition, the understanding of the moxibustion radiation spectrum is inconsistent. Generally, the peak value is 0.8∼5.6 m. Some researchers believe that moxibustion can produce infrared radiation energy that penetrates the skin, reaches the muscle tissue, and resonates with human acupoints, which may be one of the mechanisms of moxibustion. Therefore, attention should be paid to the spectral range of the light effect and the mechanism of wave peak in the treatment of dysmenorrhea to provide the experimental basis for the target of moxibustion in the treatment of PD [99, 100].

### 5.3. Moxa Smoke

Moxa smoke produced by moxibustion contains more than 200 chemical components [101]. Moxa smoke has anti-inflammatory effects, promotes blood lipid metabolism, improves immune function, has antibacterial and antitumor effects, and has a particular analgesic effect, which may be one of the mechanisms of moxibustion in the treatment of PD. From the 3-year-old *A. argyi* Ci-li et al., certain analgesic biological substances in it could have a beneficial effect on the human body [102]. Meizhen et al. [103] found that *A. argyi* leaves were rich in flavonoids, which had a sound effect on analgesia, promoted blood circulation, and had immunosuppression effects. The analgesic substances contained in moxa smoke may be related to their effect on pain in PD treatment.

### 5.4. Drug Effect

Wormwood leaves are warm in nature, can warm meridians, disperse cold and relieve pain, and enhance immunity, hemostasis, and anticoagulants, activating complement, antibacterial, anti-inflammatory, antiviral, and antioxygen free radicals [104]. Partitioned moxibustion adds ginger, garlic, or traditional Chinese medicine based on moxibustion to play the role of warming meridians and dispersing cold and conventional Chinese medicine. Ginger, garlic, or traditional Chinese medicine is placed on human acupoints with thin cuticles. Under the action of moxibustion heat, material-partitioned moxibustion absorbs the drugs into the human body through the skin and capillaries at a specific rate to play the role of drugs [105].

## 6. Summary and Outlook

In summary, PD is the dominant disease in moxibustion. Various moxibustion methods have been widely used in specific treatment methods, such as thermal, thunder-fire, partitioned, and spreading moxibustion. This paper expounds on the mechanisms of heat effect, light effect, moxa smoke, and drug effect of moxibustion and research on the treatment of PD by moxibustion by regulating endocrine hormones, improving immune function, regulating nerve factors, and improving uterine microcirculation. The above mechanism has been preliminarily clarified, but there are still problems that need to be further studied. The objective visual research evidence provided by neuroimaging suggests that moxibustion can not only have an analgesic effect in the short term but also have an excellent long-term analgesic effect in the treatment of PD. The brain regions that might be analgesia included the right fusiform gyrus, left horn gyrus, left dorsal lateral superior frontal gyrus, right middle frontal gyrus, left middle occipital gyrus, anterior cingulate gyrus, left lateral cingulate gyrus, and right anterior central gyrus. There are two possible circuits for moxibustion to relieve pain in PD: the frontal cortex basal ganglia cerebral cortex, with the lenticular nucleus as the critical component. The second is the parietal basal ganglia marginal frontal lobe. Both pathways can promote the expression of goal-directed behavior through emotional regulation.

Previous studies have the following limitations. First, moxibustion is effective in treating PD, but it is not clear which mechanism is effective for moxibustion. Second, there have been few studies on the spectral range and wave peak of moxibustion in recent years, and there is no unified conclusion. Third, studies on the mechanism of moxibustion for PD mainly focus on endocrine factors, whereas studies on other mechanisms are relatively few. Finally, the application of neuroimaging technology is lacking or single, and there is a lack of relevant research evidence. The number of research samples is small, which requires more experimental data as support. The analysis angle is mostly one or two types of low-frequency amplitude, local consistency, and brain function connection, but there is a lack of systematic and comprehensive data discussion.

## 7. Future Research Suggestions

The research direction of moxibustion in the treatment of PD should first clarify the relationship between the efficacy of moxibustion in the treatment of PD and related factors, such as acupoint moxibustion duration, moxibustion volume, and drug selection. To determine the effective radiation spectrum range and wave peak of moxibustion in the treatment of PD to provide an experimental basis for selecting radiation materials for the research and development of wearable devices for PD treatment, positron emission tomography/X-ray computed tomography, transcranial magnetic stimulation, electroencephalogram, magnetoencephalography, and other neuroimaging methods should be used. The study's sample size needs to be increased to ensure that the evidence is more objective, and the angle of data analysis should include low-frequency amplitude, local consistency, and brain function connection. Future studies should focus on multimodal neuroimaging technology and improve the brain response mechanism of moxibustion in the treatment of PD as soon as possible.

## Figures and Tables

**Table 1:** Overview of clinical studies of moxibustion.

Intervention method	Number of patients with PD	Experiment method	Observation of efficacy	Result
The herb-partitioned moxibustion [73]	171	Herb-partitioned moxibustion group (A) and starch-partitioned moxibustion group (B) were applied to shenque (CV8) (umbilical every menstrual cycle approximately 2∼3 times, until menstruating). Acupuncture group (C) was provided at sanyinjiao (SP6) (started acupuncture 3∼5 days before menstruation, once a day until menstruating). Three menstrual cycles are considered a course of treatment.	Clinical efficacy, E2, P, PGF2*α*	The cured rate in group A was better than those in groups B and C (*P* < 0.05). In group A, E2 and PGF2*α* levels were decreased, and P level was increased. In groups B and C, PGF2*α* levels were reduced. The results in group A were better than those in groups B and C (*P* < 0.05).

Heat-sensitive sensation and the conventional warm sensation of moxibustion [74]	189	Heat-sensitive moxibustion and conventional warm sensation groups were applied to guanyuan (GV4) for 40 min beginning 5 days before menstruation. Each menstrual cycle was treated for (7 ± 2) days, and both groups were treated for three periods.	MPQ, CMSS	MPQ and CMSS scores were lower in the treatment group than the control group (*P* < 0.01).

Ginger moxibustion combined with acupuncture and ibuprofen sustained-release capsules [75]	60	The treatment group was treated with ginger moxibustion at shenque (RN8) cooperated with acupuncture at sanyinjiao (SP6), zusanli (ST36), hegu (LI4), and neiguan (PC6) once per day, starting 5 days before menstruation continued for 7 days in each menstrual cycle. The control group took orally two times/day when symptoms of dysmenorrhea occurred. Both groups were treated for three menstrual cycles.	Effective rate, VAS score, PGE2, PGF2*α*	The instant curative effect, recent curative effect, and long-term curative effect in the treatment group were better than those in the control groups (*P* < 0.05). The long-term VAS score and PGE2 and PGF2a levels in the treatment group were better than those of the control group.

Moxibustion [76]	147	Moxibustion was applied for 20 min at guanyuan (GV4) once daily, seven times in total. Staring 5 days before menstruation, continued for 7 days for three consecutive menstrual cycles, followed up for three menstrual cycles.	The practical clinical rate, TCM symptoms, VAS score, CMSS score, persistent pain time, usage of painkillers, and QOL	The total effective rate was 44.89%, the total score of the symptoms, VAS score, CMSS score, pain persistent time, and usage rate of painkillers were reduced (*P* < 0.01). The scores in the perceptive field, mental field, QOL were all increased (*P* < 0.01).

Thunder-fire moxibustion combined with wenjing zhitong decoction [77]	116	The treatment group was treated with thunder-fire moxibustion combined with wenjing zhitong decoction once per day, starting 7 days before menstruation until the end of menstruation. The control group took orally two times/day. Both groups were treated for three menstrual cycles.	Clinical efficacy, TCM symptoms, VAS score	Clinical efficacy, TCM symptoms, and VAS score in the treatment group were better than those in control group (*P* < 0.05).

Drug-spreading moxibustion and oral administration of meloxicam [78]	101	Drug-spreading moxibustion was used on the lumbosacral acupoints area and then around the lower abdominal 5 days before menstruation until the third day of menstruation, once 3 days. Meloxicam was prescribed one day before menstruation 7.5 mg at a time once a day and continuously for 3 days.	Clinical efficacy, RI, and PI	The effective rate was 92.3% in the treatment group, which was better than that in the control group (*P* < 0.05). RI and PI in the treatment group were decreased than those in the control group (*P* < 0.05).

Thunder-fire moxibustion combined with ear points and ibuprofen sustained-release capsules [79]	76	The thunder-fire moxibustion selected zhongwan (CV12), guanyuan (CV4), and double sides of zusanli (ST36) points, 30 min per point each time, once a day for 3 consecutive days. Auricular points are selected from the uterus, endocrine, shenmen (TF4), liver, and kidney. For 3 days, oral ibuprofen sustained-release capsules 0.3 g/time, once in the morning and in the evening. Both groups were treated for three menstrual cycles.	VAS score, CMSS score, PGF2*α*, and PGE2	The VAS scores and CMSS scores of the treatment group were reduced than those in the control group (*P* < 0.05). Serum PGF2*α* level was decreased, and serum PGE2 level was increased in both groups (*P* < 0.05), and the effect of the treatment group was better than that of the western medicine group (*P* < 0.05).

Herb-partitioned moxibustion at the umbilicus combined with abdominal acupuncture [80]	82	The treatment group was intervened by herb-partitioned moxibustion at the umbilicus plus abdominal acupuncture, and the control group was treated with abdominal acupuncture alone, once per day, starting 7 days before menstruation for 3 consecutive days.	Clinical efficacy, PGE2, PGF2*α*, PI, and RI	The clinical efficacy rate in the treatment group was higher than that in the control group (*P* < 0.05). Serum PGF2*α* level, PI, and RI were decreased, and serum PGE2 level was increased in both groups (*P* < 0.05), and the effect of the treatment group was better than that of the western medicine group (*P* < 0.05).

Moxibustion combined with warm needling [81]	120	In the control group, warm needling was used at guanyuan (CV4) and sanyinjiao (SP6). In the treatment group, besides the same treatment as the control group, moxibustion was added at shenque (CV8). The first menstrual cycle started one day before menstruation, whereas menstrual cycles 2, 3, and 4, started 3 days before menstruation, once a day for 3 days until menstruation.	The score of the severity and the score of the total frequency in the retrospective scale of dysmenorrhea symptoms, VAS score, and the safety of the two therapeutic methods.	The score of severity, score of total frequency, and VAS score of menstrual pain were all reduced, and the effect of the treatment group was better than that of the control group (*P* < 0.05). The safety evaluation was not significant between the two groups (*P* > 0.05).

Baixiao moxibustion and ibuprofen sustained-release capsules [82]	202	Patients in group A received baixiao moxibustion for 30 min; group B received baixiao moxibustion for 15 min; group C was prescribed with ibuprofen sustained-release capsules. Groups A and B were used at guanyuan (CV4), sanyinjiao (SP6), and mingmen (GV4), once per day, starting 10 days before menstruation for 7 consecutive days. Both groups were treated for three menstrual cycles.	The real-time, short-term, and long-term VAS score, RI, PI, PSV, EDV, and PGF2*α*	The treatment group's real-time, short-term, and long-term efficacy were better than those of the control group (*P* < 0.05). The VAS score, RI, PI, and PGF2*α* levels were decreased, and PSV and EDV were increased after treatment in the three groups. Both the effects of the treatment group were better than those of the control group (*P* < 0.05).

PGE2, prostaglandin E2; PGF2*α*, prostaglandin F2 *α*; P, progesterone; E2, estradiol; VAS, visual analog scale; CMSS, cox menstrual symptom scale; TCM symptoms, traditional Chinese medicine syndrome factors; MPQ, mcgill pain questionnaire; QOL, quality of life. Uterine-artery hemodynamic indexes: resistance index (RI), pulsatility index (PI), peak systolic velocity (PSV), end diastolic velocity (EDV).

## Data Availability

The data used to support the findings of this study are included within the article.
